# Myocardial Proteomics Based on Smart Fog Computing and Its Application in Sports

**DOI:** 10.1155/2022/1471916

**Published:** 2022-02-02

**Authors:** Fucai Zhang, Yejin Wu

**Affiliations:** School of Physical Education and Health, LinYi University, LinYi 276000, China

## Abstract

How to strengthen physical fitness to improve the effect and efficiency of sports is an important research direction worthy of research. In response to these problems and limitations, Smart Fog Computing technology is introduced in this paper. Taking rats as the research object, the effective quantitative analysis and research of aerobic exercise on myocardial proteome are achieved through combining the business scope of myocardial proteomics, and connecting corresponding continuous aerobic exercises, verified by simulation analysis. The simulation research results show that the smart fog calculation is effective. For moderate-intensity aerobic exercise, the expression and intensity of the corresponding myocardial protein are changed significantly, and the corresponding heart becomes larger; meanwhile, moderate aerobic exercise can improve the metabolism and enhance digestive ability.

## 1. Introduction

With the continuous development of social economy, sports are increasingly valued and favored by people [1, 2]. However, because people have different physiques and cannot become sports athletes, for ordinary people, it can be achieved through corresponding food or nutritional supplements [3, 4]. It should be noted that some scholars have noticed the influence of related factors and then concentrated on the influence of myocardial mitochondrial protein [5, 6]. The emergence of aerobic exercise can gradually improve the related energy metabolism such as synthase in the corresponding myocardial protein tissue, further save the corresponding heart function, improve the corresponding pumping function, and accordingly meet the corresponding exercise needs. For different cells, the types of proteins are different under different pathological or physiological conditions [7, 8]. Therefore, the proteomics requires to be analyzed in terms of comprehensive and holistic analysis of dynamic changes in cells, so as to analyze protein composition, expression, modification, and other states, further analyze the relationship between proteins, and reveal the laws of proteomics and cell activity [9, 10].

However, it should be noted that, with the increase of amount of exercise and practice, different exercise loads will be achieved, which will induce corresponding differences in the expression characteristics of the myocardial proteome, and thus discover different physiological and physical loads, which can achieve the differential expression of myocardial proteomics from the various factors such as load density and continuous training period [11, 12]. Therefore, it can be seen that exercise load may guide the recombination of myocardial proteomics to achieve the reshaping of the shape, state, and structure of the heart. In response to these needs and limitations, smart fog calculations is introduced in this paper, and the effective quantitative analysis and research of aerobic exercise on myocardial proteome are realized through combining the effects of aerobic exercise under a certain intensity on myocardial proteomics and taking rats as the research object, connecting corresponding continuous aerobic exercises in series, which is verified by simulation analysis, aiming to quantitatively analyze the influence law of myocardial proteomics.

## 2. Application Strategy of Motion Feedback System Based on Smart Fog Computing

The principle of network operation situation evaluation is to obtain the evaluation value of the current network operation situation through inference calculation based on the acquisition of operation data of various equipment and network operation and maintenance data, so as to reflect the overall operation of the network. From a mathematical point of view, the network operation situation evaluation is a mapping from the impact factor to the result value, and its mathematical model is

(1) $$\begin{array}{c} \text{SA}=f\left(x_{1},x_{2},\ldots ,x_{m}\right). \end{array}$$

In the formula, SA represents the network situation evaluation value, and the value is taken as [0, 100]; *x*_*i*_ is the influencing factor of network operation, that is, the evaluation index, where *i* = 1, 2,…, *m*; *f* is the specific evaluation method, which is the realization of quantitative assessment of network operation situation proposed in this article based on the improved BP neural network model of entropy method and LM algorithm.

**Figure 1:**
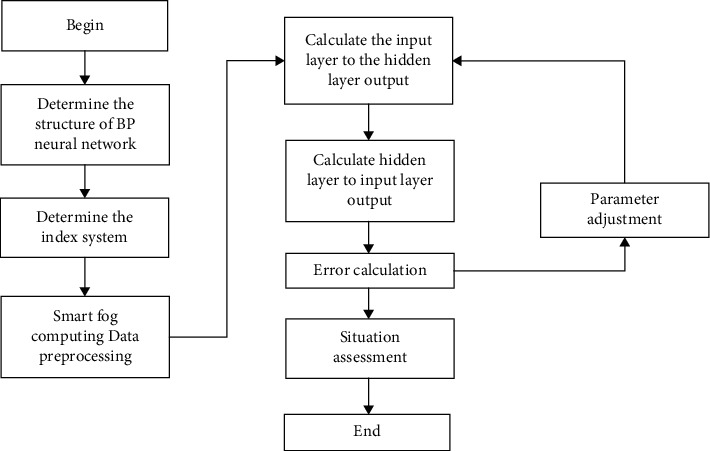
Motion feedback evaluation process based on Smart Fog Computing.

The entropy method and the Smart Fog Computing improved by the L-M algorithm are used in this paper to conduct the training process of situation assessment as shown in Figure 1.

The steps of determining the BP neural network structure, determining the index system, data preprocessing, and calculation of the index weight through entropy method as the model's initial parameters, BP neural network training model, and LM algorithm modification parameters are carried out successively. If the model error or the number of iterations meets the expected value, then the model training ends.

**Figure 2:**
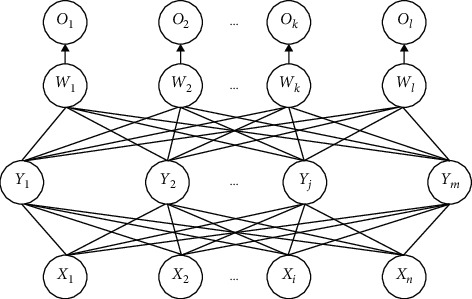
Three-layer BP neural network model.

The square sum of the output resulting errors is taken as the objective function in the network model, and the parameter value is adjusted according to the gradient descent method to reduce the error. The model structure is shown in Figure 2.

Take the network situation assessment as an example.

(2) $$\begin{array}{c} y_{j}=f\left(\sum_{i=1}^{n}v_{ij}x_{i}-\theta_{j}\right). \end{array}$$

In the formula, the function *f* is the selected activation function, which is usually expressed with the sigmoid function as

(3) $$\begin{array}{c} f\left(x\right)=\frac{1}{1+e^{-x}}. \end{array}$$

Similarly, for the *k*th neuron in the output layer, its output is

(4) $$\begin{array}{c} o_{k}=f\left(\sum_{j=1}^{m}w_{jk}y_{j}-r_{k}\right). \end{array}$$

The error of the calculation result is expressed by the least square method:

(5) $$\begin{array}{c} E_{k}=\frac{1}{2}\sum_{k=1}^{l}{\left(o_{k}-d_{k}\right)}^{2}. \end{array}$$

The above is the forward calculation process.

If the error does not meet the expected value, totaling (*N* + *L*) × *M* + *M* + *L* parameters. This process is a reverse calculation process.

In the reverse calculation process, the partial derivative of each parameter that needs to be adjusted is calculated, the parameter value is changed according to the gradient descent method, and the learning rate *η* is set to control the range of variation of parameter. *w*_*jk*_ is taken as an example, and the weight adjustment value is

(6) $$\begin{array}{c} \Delta w_{jk}=-\eta \frac{\partial E_{k}}{\partial w_{jk}}. \end{array}$$

Realization of multiple iterations of forward and reverse directions is needed for Smart Fog Computing, and the number of iterations or allowable error range is set as the iteration termination condition to obtain the final model [13, 14].

The nonlinear mapping ability of Smart Fog Computing is extremely powerful, which can solve many practical application problems, and the network structure is flexible; but there are also some defects, such as slow learning speed, prone to fall into local optimal solutions, etc. These deficiencies may cause the lower accuracy and longer training time of situation assessment model based on the standard BP neural network. In response to these problems, an improved method based on Smart Fog Computing is proposed in this paper.

In the situation assessment method based on Smart Fog Computing, the final training effect of the BP neural network model is affected by the initial value of the model parameters. The index data of the network situation assessment is imported by the BP neural network model, so the index weights optimized by the entropy method are selected as the initialization parameters of the model.

To use the entropy method to calculate the index weight, the index system must first be determined.

For a total of *n* sample data and *m* indicators required for situation assessment, the *j*-th indicator value of the *i*-th data is denoted as *x*_*ij*_. The normalized definition of data is shown in

(7) $$\begin{array}{c} x_{ij}^{\prime }=\frac{x_{ij}-\min\left(x_{j}\right)}{\max\left(x_{j}\right)-\min\left(x_{j}\right)}. \end{array}$$

For convenience, the data *x*_*ij*_′ after normalization is still recorded as *x*_*ij*_.

Calculate the proportion of the *i*-th sample in the *j*-th index as

(8) $$\begin{array}{c} p_{ij}=\frac{x_{\text{ij}}}{\sum_{i=1}^{n}x_{ij}}. \end{array}$$

The calculation of entropy is

(9) $$\begin{array}{c} e_{j}=-k\sum_{i=1}^{n}p_{ij}\ln\left(p_{ij}\right). \end{array}$$

In the formula, *k*=1/ln  *n* satisfies *e*_*j*_ ≥ 0.

Calculate the information entropy redundancy of the *j*-th index as

(10) $$\begin{array}{c} d_{j}=1-e_{j}. \end{array}$$

Calculate the weight of the *j*-th index as

(11) $$\begin{array}{c} w_{j}=\frac{d_{j}}{\sum_{j=1}^{m}d_{j}}. \end{array}$$

Smart Fog Computing can deeply reflect the distinguishing ability of indicators, determine the weight of indicators, and have high credibility and accuracy. In the network situation assessment problem, the index weight calculated is used as the initial value of the parameters, which improves the credibility of the initial model and is conducive to the correct and rapid convergence [15].

Here, teachers can create a gamified teaching mode. Taking the 4 × 100 m relay run as an example, teachers can create different nodes on the sports field and place basketball, table tennis, badminton, boxing gloves, and other props at each node, separately before running. A separate instruction is issued for each club student. The instruction can include the name of a well-known athlete in a certain sport, sports rules, etc., so as to guide students to choose props based on the instruction and put them in the finish basket after finishing the run. The heartbeat, breathing rate, speed, and other data detected in the motion feedback system are scored as well as the correctness and wrong results of the instructions, which not only helps improve the fun of running training, but also realizes the training of students' basic sports knowledge and cultivates students' teamwork awareness and further improve students' enthusiasm for sports and enthusiasm for competition.

For sports, teachers are often accustomed to guiding traditional running and gymnastics to participate in collective exercise. However, due to the different physical fitness of students, the students are not often effectively supervised and guided by this model. Therefore, students' exercise is often conducted just in the classroom, but it cannot achieve the exertion of effect of sports. With the continuous development of technologies such as the Internet of Things, new technologies such as smart fog computing can fully cover and extend the corresponding smart watches and smart bracelets to achieve the full coverage and extension of the monitoring scope of sports. Meanwhile, big data analysis can be used to summarize the overall exercise behavior of students.

Big data intelligent analysis technology can generate instructive exercise information by processing the collected raw exercise data and provide an effective reference for the adjustment of the teacher's teaching plan and the setting of teaching priorities. Taking badminton training as an example, teachers can obtain information about different students' movement postures, scores, hitting techniques, and sports area routes in the functional modules of the sports feedback system and use big data intelligent analysis technology to obtain the internal information between different indicators. Associate and then realize the quantification processing and calculation of sports information, presenting an intuitive evaluation of sports effects. Teachers need to use the exercise effect evaluation to judge the students' deficiencies in sports skills and technical programs and use this as a benchmark to adjust training content and methods, provide students with more professional and effective teaching guidance, and improve their sports skills and communicate effectively. At the same time, teachers can also use big data technology to comprehensively analyze the students' multiple training results and extract the progress curve around their original exercise level, which can be used as the benchmark for the evaluation of students' sports performance, so as to better improve the scientific nature of the evaluation of physical education and promote students' self-confidence in sports and the development of good exercise habits.

## 3. Application of Motion Feedback System in Middle School Physical Education Teaching

### 3.1. Promoting the Construction of Physical Education Informatization

For sports feedback, it is a kind of intelligent supervision that gathers big data analysis and intelligent IoT, which can effectively analyze sports behavior data and realize real-time or quasi-real-time analysis and monitoring of indicators, thus forming corresponding sports feedback forms, to realize dynamic analysis and evaluation during sports training, thereby reducing the cost of students' exercise analysis and promoting the in-depth application of smart fog computing.

### 3.2. Educational Requirements for Implementing Classified Guidance

The corresponding feedback system can be used to realize the collection, sorting, processing, analysis, and decision support of different individual motion modes, exercise speed, and other motion data, provide the corresponding teachers with personalized motion programs, and provide individual targeted guidance in different categories, thereby improving the overall effectiveness of physical education.

### 3.3. Promoting the Effective Improvement of Students' Physical Quality

The current sports feedback system is mainly embodied as wearable smart devices, including sports bracelets, smart wrist watch, and VR glasses, with functions such as exercise step counting, heart rate monitoring, and GPS positioning, to achieve effective capture of data on student movement trajectories, exercise time, etc. Physical education teachers can use their management authority to obtain the exercise data of different students, use big data intelligent analysis technology to collect the students' heart rate, speed, breathing frequency, and other parameters during exercise, and, at the same time, can judge whether the student's exercise is in place and whether the trajectory is in place. Meet the requirements, in order to provide students with personalized guidance, with the help of the normative guidance of sports behavior, to better promote the effective improvement of students' physical fitness.

## 4. Simulation Experiment

### 4.1. Heart Weight Changes

**Figure 3:**
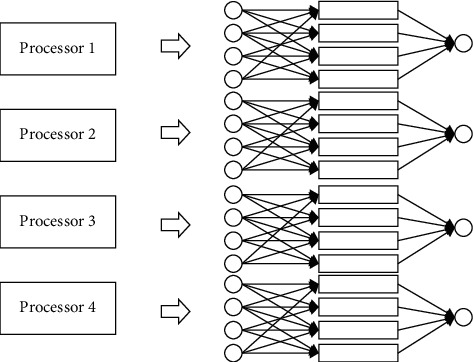
Data parallelization process.

**Figure 4:**
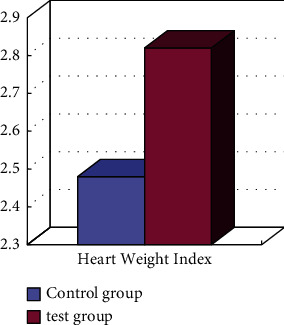
Changes in heart weight index measurement.

In order to verify the effectiveness of smart fog computing, the corresponding fault diagnosis model is needed to be collected and constructed, and training can be conducted according to the corresponding data, and finally the online detection is realized in this paper. The specific fault diagnosis is shown in Figure 3:

Sports feedback is integrated; taking rats as an example, the results of their sports feedback are comprehensively compared, as shown in Figure 4. From the results, it can be seen that the heart weight and heart weight index of big data have corresponding changes, so you can see that, after a certain period of aerobic exercise, the rat's heart has undergone certain changes.

### 4.2. Mass Spectrum Identification Result

**Figure 5:**
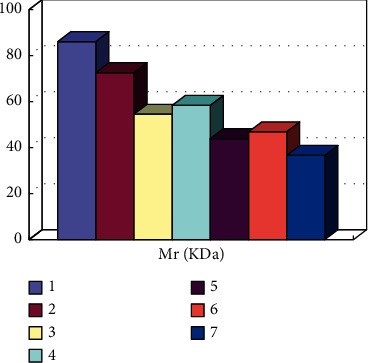
Mass spectrometric identification of target protein spots related to myocardial energy metabolism.

After a certain period of aerobic exercise, changes in the expression of myocardial protein in rats occur. Therefore, it is necessary to connect in series and use corresponding instruments for detection and identification to achieve quantitative analysis. As shown in Figure 5, it can be seen from the results that protein 7 is related to the metabolic energy of the myocardium.

### 4.3. Analysis of Changes in Heart Weight

Through corresponding experimental comparison, it can be seen that the rat's heart weight and heart weight index have increased. Therefore, the change of heart weight is used to analyze in this paper, and it can be obtained that, after a certain period of moderate aerobic exercise, the increase of myocardial physiology can be achieved, which results in a difference in the energy metabolism of some myocardial proteomics, which leads to an increase change in the level of myocardial metabolism, achieving myocardial contraction.

### 4.4. Analysis of Mass Spectrum Identification

In the process of heart remodeling during exercise, the pathway of cardiac energy metabolism has undergone corresponding changes. These proteins are mainly related to the tricarboxylic acid cycle and amino acid metabolism process in the aerobic oxidative metabolism of the myocardium.

The effect of moderate-intensity aerobic exercise on atrial myocardial tricarboxylic acid cycle: the expression of ACO2 may have expression difference of myocardium in different positions in different intensities, different continuous training periods, and different positions. The mechanism of its differential expression needs further experimental study. This experiment may increase the expression of aconitate hydratase through 4 weeks of moderate-intensity aerobic exercise, so as to improve the energy supply of the heart and enhance the contractility of atrial muscle.

**Figure 6:**
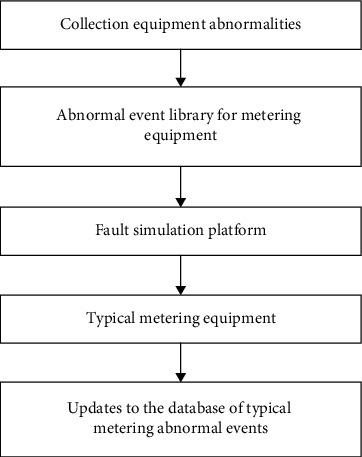
Establishment and verification of abnormal event database.

First, collect the list of abnormal events in gateway measurement, and establish a database of abnormal events in metering equipment. Common abnormal events include electric energy meter error exceeding tolerance, PT secondary voltage drop exceeding tolerance, secondary circuit pressure loss, current loss, phase failure, and secondary load overrun, unbalanced current, overlimit demand, abnormal line loss, and unbalanced bus. Then, test typical measurement equipment, and verify the system's identification through the laboratory failure simulation platform to simulate various measurement abnormal events. According to the test results, the system's functions of distinguishing, recording, and alarming abnormal measurement events are verified, and meanwhile, the judgment rules and thresholds of abnormal measurement events are improved and standardized (Figure 6).

The mitochondrial succinate dehydrogenase coenzyme flavin subunit in atrial muscle declined by 7 times, and the expression of cytoplasmic malate dehydrogenase declined by 7.2 times after exercise. The difference in experimental results may be related to many factors. Therefore, a certain period of aerobic exercise can promote the increased expression of the myocardial protein of pyruvate dehydrogenase El*α*1, realize the supply of heart energy, and enhance the contraction and pumping ability of the heart.

In this experiment, the mitochondrial succinate dehydrogenase coenzyme flavin subunit in atrial muscle was downregulated by 6.9 times, and the expression of cytoplasmic malate dehydrogenase was downregulated by 7.3 times after exercise. The difference in experimental results may be related to the intensity of exercise. The duration of exercise is related to the structure and function of myocardial tissue in different parts. The main function of the atrial muscle is not to contract and pump blood. Its ejection process is short, and the contraction work value is smaller than that of the ventricular muscle. In addition, there was a 4-week 60%–70% VO2max moderate-intensity aerobic exercise due to low exercise intensity and short exercise duration. It may be that the body is in the stage of exercise adaptation and may not produce strong oxidative stress on atrial muscle cells. Stimulate the expression of malate dehydrogenase and mitochondrial succinate dehydrogenase coenzyme flavin subunits of atrial muscle, but the reasons for the decrease in expression level need to be further explored and verified:

Point 265 is identified as pyruvate dehydrogenase El*α*1, which belongs to the pyruvate dehydrogenase complex system. Pyruvate dehydrogenase complex (PDHc) is the key enzyme system that catalyzes the oxidation and decompression of ketone acid to form acetyl-CoA and plays an important role in the body's aerobic oxidation metabolism process.

The expression of pyruvate dehydrogenase El*α*1 was “absent” in the right ventricular muscle of rats after 4 weeks of moderate-intensity aerobic exercise (70%–80% VO2max). There was no difference in its expression after 8 weeks of exercise, and its expression after 12 weeks of exercise. The expression level was upregulated by 33.3 times. The expression of this protein has a time effect. As the body slowly adapts to exercise intensity, it finally shows a high expression of adaptability to improve the myocardial oxidative metabolism capacity. Studies have found that the protein expression in right ventricular muscle is upregulated by 5.3 times after 8 weeks of exercise (60%–70% VO2max aerobic). In the results of this experiment, 4 weeks of moderate-intensity aerobic exercise induced an upregulation of the protein expression in atrial muscle and left ventricular muscle by 6.4 and 5.8 times, respectively. Based on the above research results, long-term moderate-intensity aerobic exercise can promote pyruvate dehydrogenase El*α*1 increased expression in various parts of myocardial tissue and, as a result, accelerates the oxidative decompression of pyruvate in the body, provides sufficient acetyl-CoA for the myocardial triacyl acid cycle process, improves the energy supply of the heart, and enhances the contraction and pumping ability of the heart.

The effect of moderate-intensity aerobic exercise on the amino acid metabolism of atrial muscle: point 393 is identified as isovaleryl-CoA dehydrogenase. It is involved in the metabolic decomposition of leucine, an important catalytic enzyme for leucine metabolism. Point 236 is identified as mitochondrial dihydrolipoic acid dehydrogenase, which belongs to the multienzyme complex of glycine dehydrogenase and reductase and participates in the metabolism of glycine. In addition, it also works with pyruvate dehydrogenase and dihydrolipoic acid acetyltransferase. It constitutes the pyruvate dehydrogenase complex and belongs to the FAD-dependent enzyme.

After an acute exhaustive exercise intervention, isovaleryl-CoA dehydrogenase is “absent” expression in the atrial muscle, which may cause the accumulation of isovaleric acid, a metabolite of leucine, in the process of leucine degradation, and make the intracellular pH. When the value increases, the energy metabolism of myocardial cells is impaired, and the myocardial contractility decreases. This experiment found that 4 weeks of moderate-intensity exercise reduced the expression of isovaleryl-CoA dehydrogenase in atrial muscle by 6.9 times, and the expression of mitochondrial dihydrolipoate dehydrogenase by 5.7 times. The oxygen demand is great, and the energy supply process of mature cardiomyocyte activity is mainly supplied by the aerobic oxidation of sugar, and the energy supply ratio of amino acid is very small. Hafstad's research shows that moderate-intensity treadmill exercise (65%–70% VO2max) improves the utilization of rat myocardial glucose and improves the aerobic capacity of the body. Therefore, the 4 weeks of moderate-intensity aerobic exercise in this study does not require a large amount of amino acid mobilization. It may be so in order to save the body's amino acids and protein. After exercise, the expression of the above two proteins in atrial muscle was inhibited. In this experiment, the downregulation of the expression of mitochondrial dihydrolipoate dehydrogenase may also be related to the upregulation of pyruvate deoxygenase El*α*1. In order to optimize the energy supply structure, the upregulation of pyruvate deoxygenase El*α*1 competitively inhibited dihydrolipoic acid dehydrogenation. For enzymes, the relationship between them in the metabolism of cardiomyocytes needs to be further explored.

The number point is identified as methylmalonate semialdehyde dehydrogenase [acyl], which plays a role in the metabolism of glycine and HI and belongs to the dehydrogenase family. There is no in-depth study of this protein in the field of sports medicine research: in this experiment, it was found that the expression level of this protein was upregulated by 6.3 times after 4 weeks of moderate-intensity aerobic exercise, which may bring new research content to the study of exercise to improve myocardial material and energy metabolism.

At present, with the continuous development of proteomics research technology, more and more researchers have applied proteomics and related technologies to the research of cardiovascular diseases and have made good progress, involving the expansion type proteomics research on cardiomyopathy, acute coronary syndrome, heart failure, hypertension, coronary heart disease, atrial fibrillation, myocardial infarction, vascular disease, and hyperlipidemia.

The research of sports heart proteomics is still in a preliminary stage of exploration. In recent years, the research on this topic is gradually increasing and deepening. The results of the study found that high-intensity exercise caused significant differences in the expression and quality of the proteome of rat myocardium. The effect of high-intensity exercise on the differential expression of the proteome of atrial and ventricular muscles in rats and exercise is studied. The duration of the load is related. With the extension of the duration of the exercise load, the rat's myocardial proteome changes adaptively, and the expression of more and more types and numbers of protein spots changes adaptively.

In this study, moderate-intensity aerobic exercise during a certain period of cycle does not require a large amount of amino acid to be mobilized for energy. After exercise, the expression of the above two proteins in atrial muscle probably was inhibited in order to save amino acids and protein in the body.

The research of sports heart proteomics is still in a preliminary stage of exploration. In recent years, the research on this topic is gradually increasing and deepening. Moderate-intensity aerobic exercise can make good adaptability changes in the heart. The study of the myocardium under this exercise intensity from the molecular level of protein can provide a theoretical basis for explaining the related mechanism as a whole. The changes in the myocardial proteome caused by long-term moderate-intensity exercise mainly occurred in the first 8 weeks, and different myocardial parts showed different differential expression characteristics.

**Figure 7:**
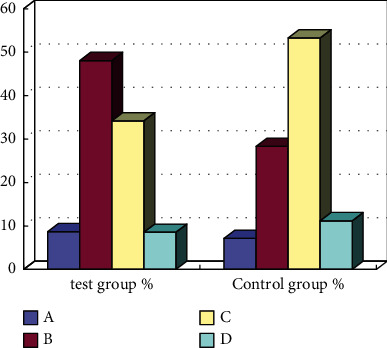
Comparison of physical fitness classification of middle school students after the intervention and during the backtest.

Judging from the physical fitness classification of posttest and backtest, the physical fitness test results of the experimental group rats showed a phenomenon that the concentration of the rats in the test group rose from the “pass” level to the “good” level before the intervention, while, in the control group rats, all three physical fitness test results showed that they were gathered at the “pass” level (Figure 7). This also shows that intervention education has a good effect on improving the physical fitness of rats.

**Figure 8:**
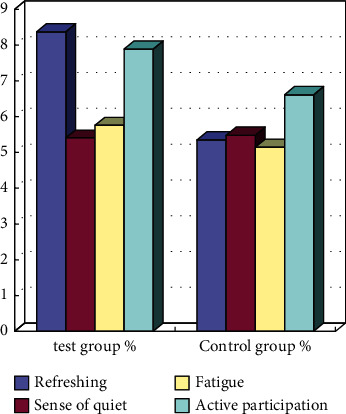
Comparison of changes in rats' physical exercise sensation after intervention and during backtesting.

The exercise feeling test after the intervention showed (see Figure 8) that the comparison of the two dimensions of refreshment and active participation in the experimental group of rats was significantly higher than that of the control group (*P* < 0.05), and meanwhile, the quietness decreased, and fatigue sensation increased, but the difference between the groups was not significant (C0.05).

In this study, moderate-intensity aerobic exercise in a certain period of time does not require a large amount of amino acid mobilization for energy. It may be so in order to save amino acids and protein in the body; after exercise, the expression of the above two proteins in atrial muscle was inhibited.

The research of sports heart proteomics is still in a preliminary stage of exploration. In recent years, the research on this topic is gradually increasing and deepening. Moderate-intensity aerobic exercise can make good adaptability changes in the heart. The study of the myocardium under this exercise intensity from the protein molecular level can provide a theoretical basis for explaining the related mechanism as a whole. The changes in the myocardial proteome caused by long-term moderate-intensity exercise mainly occurred in the first 8 weeks, and different myocardial parts showed different differential expression characteristics.

Long-term aerobic exercise can improve the heart and exercise capacity of the elderly to a certain extent, which in turn promotes the improvement of the cardiovascular skills of the elderly. From the perspective of proteomics, it can be seen that moderate aerobic exercise is beneficial to the heart and can provide a certain practical basis for cardiovascular rehabilitation and treatment of the elderly. Meanwhile, moderate-intensity and continuous practice can physiologically reshape the heart, guide the heart to transform to the direction of contraction and pumping function, and finally complete the continuous expression of differentiation.

## 5. Conclusions

The continuous development of social economy has promoted people's attention to the body more and more. How to effectively carry out physical exercise and enhance nutrition is extremely important. In response to these problems and limitations, aerobic exercise monitoring is performed for a certain period of time based on smart fog computing technology, through combining the business scope of myocardial proteomics, realizing the effective analysis of aerobic exercise on myocardial proteomics, and a certain verification is performed through simulation analysis. The experimental results show that the method is effective, can promote effective metabolism, and can achieve an effective improvement of digestive ability.

## Data Availability

The labeled dataset used to support the findings of this study is available from the corresponding author upon request.
